# Independent prognostic value of angiogenesis and the level of plasminogen activator inhibitor type 1 in breast cancer patients

**DOI:** 10.1038/sj.bjc.6600662

**Published:** 2003-01-28

**Authors:** S Hansen, J Overgaard, C Rose, Ann Knoop, A-V Lænkholm, J Andersen, F B Sørensen, P A Andreasen

**Affiliations:** 1 Department of Oncology, Oncological Research Centre, Odense University Hospital, DK-5000 Odense C, Denmark; 2 Department of Experimental Clinical Oncology, Aarhus University Hospital, DK-8000 Aarhus C, Denmark; 3 Department of Oncology, Aarhus University Hospital, DK-8000 Aarhus C, Denmark; 4 Department of Pathology, Aarhus University Hospital, DK-8000 Aarhus C, Denmark; 5 Department of Oncology, Lund University Hospital, S-221 85 Lund, Sweden; 6 Department of Molecular and Structural Biology, Aarhus University, DK-8000 Aarhus C, Denmark

**Keywords:** breast-neoplasms, neovascularization, prognosis, survival-analysis

## Abstract

Tumour angiogenesis and the levels of plasminogen activator inhibitor type 1 (PAI-1) are both informative prognostic markers in breast cancer. In cell cultures and in animal model systems, PAI-1 has a proangiogenic effect. To evaluate the interrelationship of angiogenesis and the PAI-1 level in breast cancer, we have evaluated the prognostic value of those factors in a total of 228 patients with primary, unilateral, invasive breast cancer, evaluated at a median follow-up time of 12 years. Microvessels were immunohistochemically stained by antibodies against CD34 and quantitated by the Chalkley counting technique. The levels of PAI-1 and its target proteinase uPA in tumour extracts were analysed by ELISA. The Chalkley count was not correlated with the levels of uPA or PAI-1. High values of uPA, PAI-1, and Chalkley count were all significantly correlated with a shorter recurrence-free survival and overall survival. In the multivariate analysis, the uPA level did not show independent prognostic impact for any of the analysed end points. In contrast, the risk of recurrence was independently and significantly predicted by both the PAI-1 level and the Chalkley count, with a hazard ratio (95% CI) of 1.6 (1.01–2.69) and 1.4 (1.02–1.81), respectively. For overall survival, the Chalkley count, but not PAI-1, was of significant independent prognostic value. The risk of death was 1.7 (1.30–2.15) for Chalkley counts in the upper tertile compared to the lower one. We conclude that the PAI-1 level and the Chalkley count are independent prognostic markers for recurrence-free survival in patients with primary breast cancer, suggesting that the prognostic impact of PAI-1 is not only based on its involvement in angiogenesis.

Tumour growth can be dependent on angiogenesis, that is, the formation of new blood vessels from the existing capillary network (Folkman, 1971, 1995). Tumour progression seems to be dependent on cancer cell controlled tissue remodelling, including angiogenesis, mediated to a large extent by the plasminogen activation system (Mignatti and Rifkin, 1996; Pepper *et al*, 1996; Mazar *et al*, 1999). The hypothesis of angiogenesis as a prognosticator has been widely investigated using different assays for determining microvessel density (Fox, 1997; Hansen *et al*, 2000a). Angiogenesis determined by Chalkley counting has shown independent prognostic value in a large population-based study of primary breast cancer patients (Hansen *et al*, 2000b). Breast cancer patients whose tumours had a Chalkley count in the upper tertile had, compared to patients belonging to the lowest tertile, a 126% increased risk of dying and patients in the middle tertile a 55% increased risk (Hansen *et al*, 2000b). Likewise, the urokinase-type (uPA) plasminogen activator system has been shown to play a crucial role in cancer metastasis (Andreasen *et al*, 1997, 2000). Various components of the uPA system, including uPA itself and its primary inhibitor, plasminogen activator inhibitor type 1 (PAI-1), have demonstrated significant prognostic impact in breast cancer patients. Thus, the level of PAI-1 in primary tumours is one of the most informative biochemical prognostic markers in several cancer types (Duffy, 1996; Harbeck *et al*, 1998; Knoop *et al*, 1998; Duffy *et al*, 1999; Look and Foekens, 1999; Janicke *et al*, 2001; Look *et al*, 2002).

In several cell culture and animal model systems, PAI-1 has been found to have a proangiogenic effect (Bajou *et al*, 1998, 2001; Lambert *et al*, 2001; McMahon *et al*, 2001; Devy *et al*, 2002). However, the clinical significance of the biological interaction of angiogenesis and the plasminogen activator system is unknown. Although the prognostic impacts of the Chalkley count and PAI-1 have been reported individually in large study populations, it is highly relevant to evaluate the combined prognostic impact of angiogenesis and the levels of the components of the plasminogen activator system in the same study population.

The aims of the present study were to evaluate the association between the Chalkley count, the levels of PAI-1 and of uPA, and the independent prognostic value of these components in relation to the classical prognostic factors in breast cancer.

## Material and methods

### Patients

The study included 228 patients who underwent surgery for primary, unilateral, invasive breast carcinoma. Inclusion was restricted to patients referred from the primary catchment area of Odense University Hospital during the period from 1 August 1984 to 1 September 1989. Mammographic screening for breast cancer was not performed in the background population during this period. Excluded were patients with distant metastasis at the time of diagnosis, locally advanced disease, inflammatory carcinoma, synchronous bilateral breast cancer, and a diagnosis of isolated carcinoma *in situ*. Women with previous malignant disease, apart from carcinoma *in situ* of the uterine cervix or nonmelanotic skin cancer, were excluded, as were women who did not undergo axillary dissection with at least one lymph node removed. The analysis was restricted to those 228 patients who had residual tumour material stored at −80°C at the time of surgery. Earlier we reported the Chalkley counts on a population-based cohort of 836 patients (Hansen *et al*, 2000b). The 228 patients are a subgroup of this cohort, sampled from a shorter period of time (432 patients), and further restricted to patients having frozen tumour material available. Also, we have earlier reported on uPA and PAI-1 from a larger cohort of 429 patients based on frozen tumour samples (Knoop *et al*, 1998). Again, the 228 patients are a subgroup of this cohort restricted to the primary catchment area of Odense University Hospital.

### Treatment

Surgery, radiotherapy, and adjuvant systemic therapy were carried out according to the nationwide recommendation of the Danish Breast Cancer Cooperative Group (DBCG) (Andersen and Mouridsen, 1988). In all, 27 patients were treated with breast-conserving surgery and all had postoperative irradiation. In total, 201 patients underwent simple mastectomy, and of these 63 had postoperative irradiation. High-risk patients (N1, T3 or T4) were offered systemic adjuvant therapy according to the DBCG programme (Fischerman and Mouridsen, 1988). In summary, 42 patients had primary chemotherapy (CMF), 50 patients primary endocrine therapy (tamoxifen), and 12 both endocrine therapy and chemo-therapy. A total of 124 patients received no systemic treatment: of these 82 were considered low-risk (N0, T1–2) patients, while 42 patients were either too old or were found to have some medical contraindications for systemic treatment.

### Follow-up

Patients were followed up on a regular basis for the first 10 years at Odense University Hospital according to the DBCG recommendations (Andersen and Mouridsen, 1988). After the first 10 years, information about recurrence was obtained by studying the clinical records from the treating department. Patients were followed until the time of the last contact or death, or until the closing date of the study, 1 March 1999. The median follow-up time was 12 years and 1/2 month.

### End points

The prognostic analysis was primarily carried out using recurrence-free survival (RFS) and overall survival (OS). The RFS analysis was based on time to first recurrence at any site including 89 events. Thus, patients who died without recurrence were censored. The OS analysis was based on death from any cause comprising 118 events.

### Histopathology

The histological type of breast tumour was determined according to the WHO guidelines (Scharff and Torloni, 1998). Histological malignancy grading followed the grading system of Bloom and Richardson (1957). Tumour size was measured as the largest diameter of the invasive carcinoma. Receptor status was retrieved from the DBCG database and considered positive if the estrogen receptor (ER) or progesterone receptor (PgR) value by the dextran-coated charcoal (DCC) method was greater than or equal to 10 fmolmg^−1^ cytosol protein. Where the DCC method was unavailable, a positive receptor status was determined by an immunohistochemical ER staining (antibody clone 1D5, DAKO, Glostrup, Denmark). ER-positive was defined as ⩾10% of the tumour cells staining positively.

### Angiogenesis

The estimation of angiogenesis by Chalkley counts has been described in detail earlier (Hansen *et al*, 2000b). Briefly, one 4 *μ*m thick section from each formalin-fixed and paraffin-embedded tumour was mounted on a ChemMate slide (DAKO, Glostrup, Denmark). Epitope retrieval was performed by microwave heating in a buffer of 10 mM Tris (pH 9), 0.5 mM EGTA. As primary antibody against CD34, we used clone QBEnd/10 (Novo Castra, Newcastle, UK), diluted 1 : 20, with overnight incubation at 4°C.

The angiogenesis assessed by the Chalkley count technique was based on scanning the tumour section at low magnification and choosing three of the most vascularised areas (hot-spots), which were assumed to have the highest number of microvessel profiles. Using a higher magnification, a 25-point Chalkley graticule was applied to each hot-spot and oriented to permit the maximum number of points to hit the immunohistochemically highlighted microvessel profiles (Leitz Ortoplane; ×250; Chalkley grid area 0.196 mm^2^). The Chalkley count was taken as the mean value of the three counts for each tumour. The Chalkley count is given as the number of hitting points without a unit; measurement range 0–25.

### uPA and PAI-1

The antigen levels of uPA and PAI-1 were assessed with ELISA as described in detail earlier (Knoop *et al*, 1998). Briefly, tissue for analysis was taken from −80°C, homogenised in a buffer of 0.1 mM Tris (pH 8.1), 0.5% Triton X-100, 10 mM EDTA, 10 *μ*gml^−1^ aprotinin (10 *μ*l buffer per mg tissue), and centrifuged. The supernatants were analysed. For the uPA ELISA, monoclonal anti-uPA IgG from hybridoma clones 2, 6, and 12 was used on the solid phase. For the PAI-1 ELISA, monoclonal anti-PAI-1 IgG from hybridoma clone 2 was used on the solid phase. The second antibody layer consisted of polyclonal rabbit anti-uPA or rabbit anti-PAI-1 antibodies. As the third layer, we used peroxidase-conjugated swine antibodies against rabbit antibodies for both ELISAs. The levels of uPA or PAI-1 were expressed as ng per mg total protein in the extracts.

### Statistics

Angiogenesis, PAI-1, and uPA were estimated without knowledge of the clinical data or prognostic outcome. Predetermined cutoff points from the earlier studies (Knoop *et al*, 1998; Hansen *et al*, 2000b) were used for the prognostic analysis: Chalkley count at 5 and 7 points, PAI-1 at 11.1 ngmg^−1^ total protein, and uPA at 4.5 ngmg^−1^ total protein. The relationship between the Chalkley count, the PAI-1 level, and the uPA level were tested by the Spearman correlation test. The possible associations of the classical clinical pathological parameters on one side and the Chalkley count, the PAI-1 level, and the uPA level on the other side were tested by the Kruskall–Wallis test. The univariate relationship between prognostic variables and the follow-up end points was illustrated by Kaplan–Meier plots of survival probabilities (Kaplan and Meier, 1958), and the survival functions were compared by the log-rank test. The multivariate relationship was evaluated by the Cox proportional hazard regression analysis (Cox, 1972). To obtain acceptable statistical strength in the multivariate analysis, it has been suggested that the number of events should be at least 10 times the total number of variables included (Harrell *et al*, 1985; Simon and Altman, 1994; Peduzzi *et al*, 1996). In this study, with a limited number of events, it is not advisable to introduce many covariates, and this was the reason for not estimating the prognostic effect of each subgroup of the variable. Therefore, the risk estimates for each variables are an average between subgroups; that is, lowest to middle to highest tertile. Thus, using eight variables in our Cox models, we assumed that we could achieve a reliable statistical strength of the risk estimates in the multivariate regression analysis. As in the earlier publication (Knoop *et al*, 1998), the Cox models included the classical prognostic factors (menopausal status, tumour size, malignancy grade, receptor status, and number of positive nodes) together with uPA, PAI-1, and the Chalkley count, in that order. The Cox models were stratified by receptor status, which did not fulfil the assumption of proportional hazard rates. Two-sided *P*-values below 0.05 were considered to be statistically significant.

## Results

### Clinical data

The tertiles of the Chalkley estimates were 5.3 and 7.0 points, of the PAI-1 levels 8.1 and 15.1 ngmg^−1^ total protein, and of the uPA levels 3.2 and 6.3 ngmg^−1^ total protein. The median (range) of the Chalkley count was 6.0 (2.7–13.0), of PAI-1 11.1 ngmg^−1^ (0.1–73.0), and of uPA of 4.7 ngmg^−1^ (0.3–21.6). Table 1

**Table 1:** Description of clinicopathological data and the association with the Chalkley count, PAI-1 levels and uPA levels

			**Chalkley count**	**PAI-1**	**uPA**
			**(points)**	**(ngmg^−1^ total protein)**	**(ngmg^−1^ total protein)**
**Variables**	***N***	**(%)**	**Median**	**Median**	**Median**
All	228	(100)	6.0	11.05	4.65
					
Age (years)					
<40	11	(5)	6.3	11.4	4.2
40–49	41	(18)	6.1	10.7	3.3
50–59	51	(22)	6.0	10.1	5.0
60–69	60	(26)	6.3	11.3	4.9
⩾70	65	(29)	5.7	11.3	5.5
			*P*=0.99	*P*=0.90	*P*=0.69
					
Menopausal status					
Pre	68	(30)	6.2	10.6	4.2
Post	160	(70)	6.0	11.3	5.0
			*P=0.86*	*P=0.31*	*P=0.27*
					
Tumour size (mm)					
⩽20	86	(38)	5.7	10.5	3.8
21–50	132	(58)	6.3	12.7	5.7
>50	10	(4)	6.5	8.1	3.0
			***P=0.004***	***P=0.019***	***P=0.004***
					
Histological malignancy grade					
I (ductal)	36	(16)	5.2	9.6	4.4
II (ductal)	87	(38)	6.0	12.1	5.4
III (ductal)	74	(32)	7.0	12.9	5.5
Other (nonductal)	31	(14)	5.1	5.1	1.8
			***P<0.001***	***P<0.001***	***P<0.001***
					
Receptor status					
Positive	174	(76)	5.7	10.8	4.5
Negative	54	(24)	7.7	12.9	5.6
			***P<0.001***	***P=0.027***	*P=0.069*
					
Lymph node metastasis					
None	101	(44)	6.0	9.7	4.2
1–3	78	(34)	6.0	12.5	5.4
⩾4	49	(22)	6.7	14.7	5.2
			*P*=0.82	***P=0.008***	*P*=0.055

Significant associations are given in bold.

shows the distributions of the clinical and pathological data of the patients in the study population and the distribution of the Chalkley count, the PAI-1 level, and the uPA level. A high Chalkley count was significantly associated with large tumour size, high histological malignancy grade, negative receptor status, but not with a high number of axillary lymph node metastases, age, or menopausal status. A high PAI-1 level was significantly associated with large tumour size, high histological malignancy grade, negative receptor status, and a high number of axillary lymph node metastases, but not with age or menopausal status. A high uPA level was significantly associated with large tumour size, high histological malignancy grade, and marginally significantly associated with negative receptor status and a high number of positive axillary lymph node metastases, but not with age or menopausal status.

### Association

Figure 1

**Figure 1:**
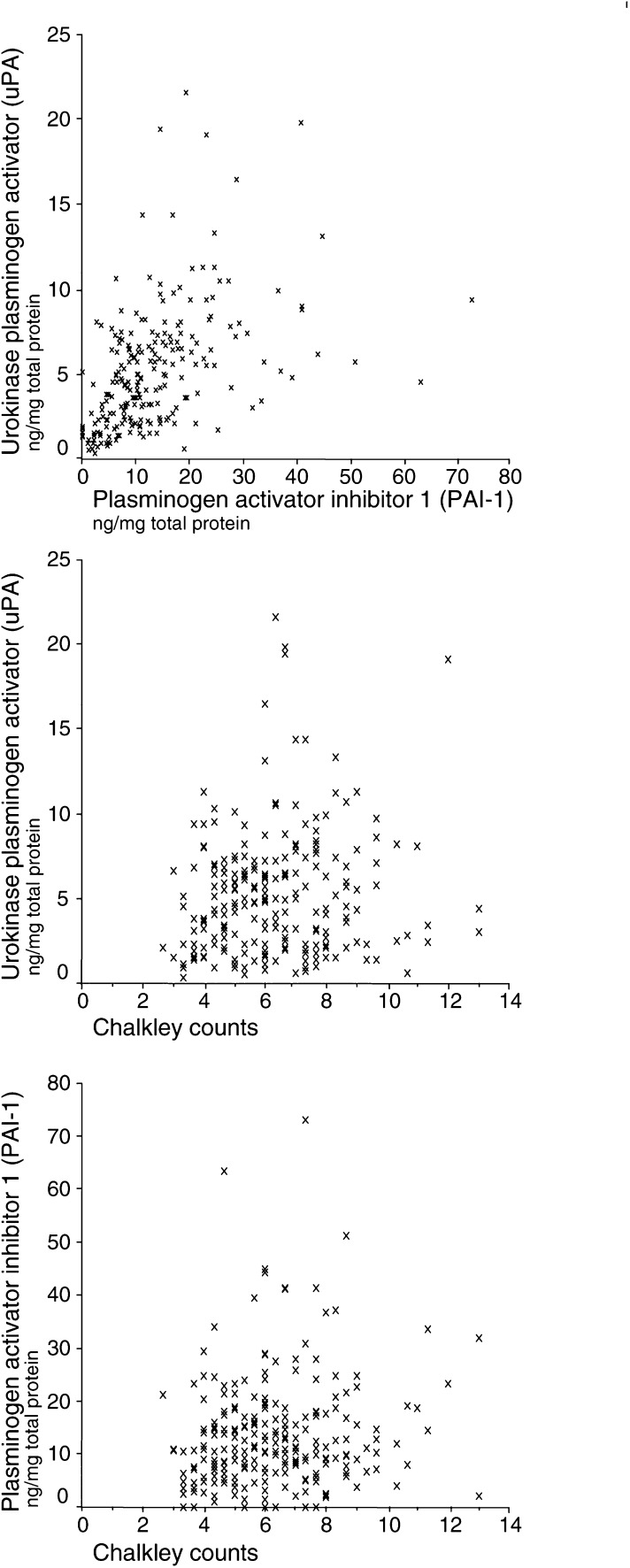
Relationships between the levels of uPA, PAI-1, and the Chalkley count.

shows scatter plots illustrating the relationship between the Chalkley count, the PAI-1 level, and the uPA level. High levels of uPA were weakly linearly associated with high levels of PAI-1 (*r*=0.57, *P<*0.001). In contrast to this, there was no indication of an association between the Chalkley count and PAI-1 or uPA levels in primary breast carcinomas.

### Univariate analysis

**Figure 2:**
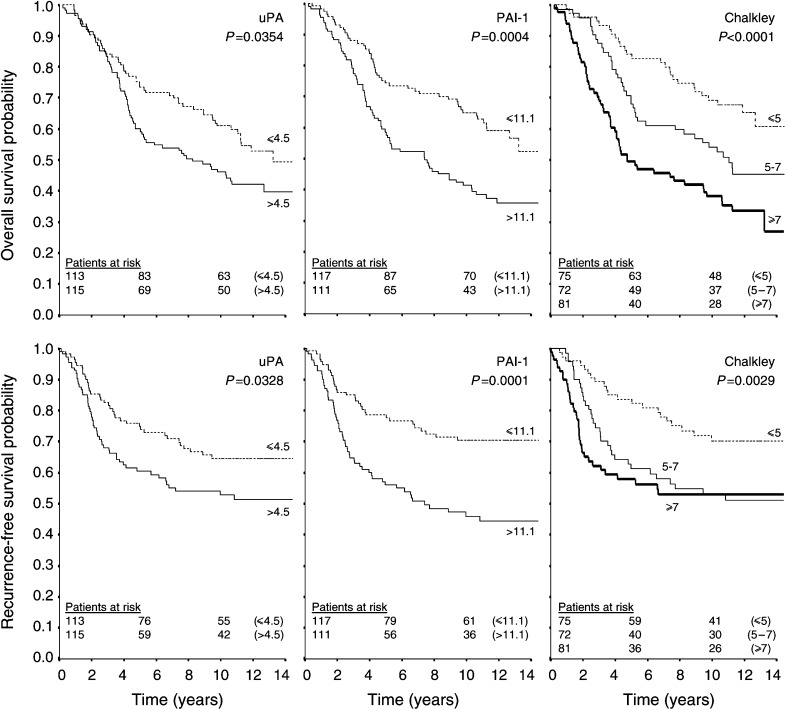
Kaplan--Meier plots of the survival probabilities for the different categories of the Chalkley count, the uPA level, and the PAI-1 level. The survival curves in the *upper row* give the probabilities of overall survival, and in the *bottom row* the probabilities of recurrence-free survival. Predefined cutoff values (see Statistics section) were 4.5 ngmg^−1^ total protein for the uPA level, 11.1 ngmg^−1^ total protein for the PAI-1 level, and 5 and 7 points for the Chalkley count. The number of patients at risk in each group is given at 0-, 5-, and 10-years follow-up.

The Kaplan–Meier plots in Figure 2

illustrate the significantly poorer prognostic outcome for patients with high Chalkley counts (*P=*0.0029, RFS; *P<*0.0001, OS), high PAI-1 levels (*P=*0.0001, RFS; *P=*0.0004, OS), and high uPA levels (*P=*0.0328, RFS; *P=*0.0354, OS). The 5-year-survival probabilities for the three investigated factors as well as the classical prognostic factors in breast cancer are tabulated in Table 2

**Table 2:** Five-year survival probabilities±s.e. in percentages for recurrence-free survival (RFS), and overall survival (OS)

		**RFS**	**OS**
**Variables**	***N***	**% (±s.e.)**	**% (±s.e.)**
All	228	68 (3)	67 (3)
			
Menopausal status			
Pre	68	67 (6)	74 (5)
Post	160	68 (4)	64 (4)
		*P*=0.926	*P*=0.011
			
Tumour size (mm)			
⩽20	86	83 (4)	81 (4)
21–50	132	59 (4)	59 (4)
>50	10	40 (15)	40 (15)
		***P=0.0004***	***P=0.0002***
			
Histological malignancy grade			
I (ductal)	36	88 (5)	92 (5)
II (ductal)	87	66 (5)	69 (5)
III (ductal)	74	55 (6)	49 (6)
Other (nonductal)	31	76 (8)	74 (8)
		***P=0.0061***	***P=0.0007***
			
Receptor status			
Positive	174	69 (4)	68 (4)
Negative	54	64 (7)	63 (7)
		*P*=0.881	*P*=0.857
			
Lymph node metastasis			
None	101	75 (4)	77 (4)
1–3	78	78 (5)	72 (5)
⩾4	49	37 (7)	37 (7)
		***P<0.0001***	***P<0.0001***
			
Chalkley count (points)			
⩽5	75	84 (4)	84 (4)
5–7	72	61 (6)	68 (5)
⩾7	81	58 (6)	49 (6)
		***P=0.0029***	***P<0.0001***
			
PAI-1 (ngmg^−1^ total protein)			
⩽11.1	117	79 (4)	74 (4)
>11.1	111	56 (5)	59 (5)
		***P=0.0001***	***P=0.0004***
			
uPA (ngmg^−1^ total protein)			
⩽4.5	113	75 (4)	73 (4)
4.5	115	60 (5)	60 (5)
		***P=0.0328***	***P=0.0354***

Significant differences in survival probabilities between the groups are given in bold.

. Among the classical prognostic factors, the univariate analysis showed a significantly poorer prognostic outcome for patients with large tumour size (*P=*0.0004, RFS; *P=*0.0002, OS), high histological malignancy grade (*P=*0.0061, RFS; *P=*0.0007, OS), high number of positive axillary lymph nodes (*P<*0.0001 for both RFS and OS), while postmenopausal patients had a significantly poorer outcome only for OS (*P=*0.011).

### Multivariate analysis

Table 3

**Table 3:** The Cox multivariate analysis estimated the hazard ratios (HR) and 95% CI for the risk of recurrence (RFS) and risk to die (OS) in a group of 228 patients with breast cancer

	**RFS**			**OS**		
**Variables**	***P***	**HR**	**(95% CI)**	***P***	**HR**	**(95% CI)**
Menopausal status	0.91	1.0	(0.61–1.55)	**0.003**	2.0	(1.26–3.12)
Tumour size	**0.015**	1.7	(1.10–2.57)	0.014	1.6	(1.10–2.23)
Malignancy grade	0.081	1.4	(0.96–1.93)	0.011	1.5	(1.10–2.09)
Lymph node status	**<0.0001**	1.9	(1.40–2.50)	<0.0001	2.0	(1.53–2.52)
UPA	0.33	1.3	(0.79–2.01)	0.60	1.1	(0.74–1.67)
PAI-1	**0.040**	1.7	(1.02–2.72)	0.28	1.3	(0.83–1.92)
Chalkley count	0.032	1.4	(1.03–1.84)	**<0.0001**	1.7	(1.32–2.20)

The variables were grouped as given in Table 2. The nonductal carcinomas were included in the group of grade II ductal carcinomas. The Cox models were stratified by oestrogen receptor status, which did not fulfill the assumption of proportional hazard rates. Bold *P*-values are given for variables with hazard ratios significantly different from one.

shows the results of the Cox multivariate regression analysis including the classical prognostic factors (menopausal status, tumour size, histological malignancy grade, receptor status, and number of axillary lymph node metastases) as well as the levels of uPA and PAI-1 and the Chalkley count, which were introduced in the model in that order. The cutoff values from Table 1 were used for the classical factors, while the cutoff values for the levels of uPA and PAI-1 and the Chalkley count were used as defined in earlier studies (Knoop *et al*, 1998; Hansen *et al*, 2000b). The nonductal carcinomas were grouped with ductal malignancy grade II carcinomas because they had approximately the same survival. Using the recurrence-free survival as an endpoint, there was significant independent information from tumour size, lymph node metastasis, PAI-1, and Chalkley count. There was a 70% increased risk of recurrence (HR=1.7 (1.02–2.72)) for patients with PAI-1 values above the median as compared to values below the median. There was a 40% increased risk of recurrence (HR=1.4 (1.03–1.84)) for patients with Chalkley counts in the middle tertiles as compared to the lowest tertile, and a further 40% increase in the risk of recurrence in having a Chalkley count in the upper tertile as compared to the middle tertile. Analysing overall survival, menopausal status, tumour size, malignancy grade, lymph node metastasis, and Chalkley counts showed a significant independent prognostic value. There was a 70% increase in the risk of dying (HR=1.7 (1.32–2.20)) for patients with Chalkley counts going from one tertile to the next.

## Discussion

Angiogenesis and extracellular proteolysis of the plasminogen activation system are of crucial importance in cancer metastasis (Andreasen *et al*, 1997). A clinical usefulness of these systems to predict the outcome in breast cancer patients has been expected. Numerous studies have established a prognostic value of the levels of uPA and PAI-1 and of angiogenesis in breast cancer patients (Fox, 1997; Harbeck *et al*, 1998; Duffy *et al*, 1999; Look and Foekens, 1999; Look *et al*, 2002). However, none of these factors are yet used in current clinical practice.

The biological interpretation of the prognostic impact of high values of uPA and of angiogenesis has been straightforward. A high number of vessel profiles should increase the nourishment and growth of the tumour and allow access of tumour cells to the circulation. High uPA levels facilitate the proteolytic degradation of the basement membranes and extracellular matrix, and thereby increase the invasive ability and metastatic potential. Moreover, angiogenesis is believed to require extracellular proteolytic activity, including uPA activity (Mignatti and Rifkin, 1996; Pepper *et al*, 1996; Mazar *et al*, 1999). The prognostic impact of high levels of PAI-1 has been more difficult to explain. Generally, we could expect a requirement for the presence of proteinase inhibitors during tissue remodelling events, because of a need to restrict proteolysis in time and space. Hence, the association of high PAI-1 levels with a poor prognostic outcome can be explained by a requirement for local downregulation of the proteolytic activity. In particular, proteolytic downregulation could support a local protection of the basement membrane surrounding the sprouting endothelial cells. This could further facilitate the capillary networking of the new tumour blood vessels. Accordingly, it has been reported that tumour angiogenesis is drastically impaired in mice with targeted disruption of the PAI-1 gene (Bajou *et al*, 1998, 2001). A few other model system studies have also suggested a proangiogenic role of PAI-1 (Lambert *et al*, 2001; McMahon *et al*, 2001; Devy *et al*, 2002). In particular, the possible role of uPA and PAI-1 in angiogenesis was of interest in the preparation of the present study. Owing to the above explanations, we had reasons to expect biological relations between levels of the components of the plasminogen activating system, especially PAI-1, and the angiogenic process. One might expect PAI-1 to be a surrogate marker for the angiogenic activity. This could be expected to be seen as a positive association between PAI-1 and the Chalkley count, and possibly a prognostic value of PAI-1 and the Chalkley count being dependent on each other.

In general, the estimates of the Chalkley count, PAI-1, and uPA from this study are in agreement with our earlier reports (Knoop *et al*, 1998; Hansen *et al*, 2000b). In the present study, the Chalkley count was not associated with the levels of uPA or PAI-1. This is in accordance with another report (Fox *et al*, 2001) showing no association between the Chalkley count and uPA or PAI-1. However, that study did not evaluate the independent prognostic value of the Chalkley count in relation to the uPA or PAI-1 levels, because of an ‘insufficient number of events for the number of variables’ (Fox *et al*, 2001). Others have reported a moderate association between angiogenesis and uPA (*r*=0.85), as well as PAI-1 (*r*=0.74) (Hildenbrand *et al*, 1995). This might be explained from the fewer (*n*=42) patients investigated in those studies, from correlating uPA from the periphery of the tumour specimen with the angiogenesis, and from estimating angiogenesis by the microvessel density method. We chose the Chalkley count estimate of angiogenesis, since this assay is somewhat less influenced by the observer variation (Hansen *et al*, 1998), and because it has a stronger prognostic value as compared to the microvessel density assay (Rose *et al*, 2000). The frozen tumour specimens used for the uPA and PAI-1 analyses do not represent the same tumour areas as the paraffin-embedded specimens used for the Chalkley counts. Owing to the tumour heterogeneity, the tumour values of biologically related factors obtained from estimates in different regions may not be associated, although measurements of the factors obtained from the same areas would be associated. The levels of PAI-1 and uPA were low in very large tumours (>50 mm) and in nonductal carcinomas. Hence, it is necessary to be aware of possible sampling errors, which in large tumours could be affected by necrotic areas, and in lobular carcinomas by the lower cellularity compared to the ductal carcinomas.

The independent prognostic effect of the Chalkley count was approximately of the same magnitude as reported earlier (Fox *et al*, 1995; Hansen *et al*, 2000b). In accordance with our earlier findings (Knoop *et al*, 1998), the uPA did not reveal any independent prognostic value. The PAI-1 estimate did have independent prognostic impact regarding the risk of recurrence as in our earlier report (Knoop *et al*, 1998). The PAI-1 estimate did not provide a significant independent prognostic value for the risk of death in the present smaller sample of the former population. This was also the case in a Cox model not including the Chalkley count (data not shown). We stratified the Cox multivariate models by receptor status, because it does not fulfil the assumption of proportional hazards. Hence, the independent prognostic estimates of uPA, PAI-1 and the Chalkley count are adjusted for the effect of the receptor status as well as the other classical prognostic factors included.

The lack of correlation between the level of PAI-1 and the Chalkley count at first sight appears in contradiction to the hypothesis of PAI-1 being implicated in angiogenesis, as discussed above. Several explanations may be offered for the lack of correlation. First, the angiogenesis may be regulated differently in the human breast tumours studied here and in the experimental models discussed above. Second, if PAI-1 is implicated in the angiogenic process, it may only be transiently expressed and its level may therefore be associated with the rate of vessel formation rather than the accumulated amount of vessels, represented by the Chalkley count (Fox *et al*, 2001). Third, it is because of heterogeneity of the level of PAI-1 and the Chalkley count within the tumour, as discussed above. Fourth, PAI-1 may have a multitude of functions in breast tumours, angiogenesis only being one among several. The latter hypothesis is favoured by the immunohistochemical localization of PAI-1 to several cell types in breast tumours, including not only endothelial cells but also fibroblasts and cancer cells (Christensen *et al*, 1996).

The major finding in our present investigation is the independent prognostic impact of both the PAI-1 and the Chalkley count in the same primary breast tumours. This fits well with the lack of association between these estimates, which may independently contribute to the regulation of tumour progression. In conclusion, both PAI-1 and the Chalkley count added significant and independent prognostic information on RFS in patients with primary breast cancer.
